# Identification and Localization of Proteins Associated with Biomineralization in the Iron Deposition Vesicles of Honeybees (*Apis mellifera*)

**DOI:** 10.1371/journal.pone.0019088

**Published:** 2011-04-26

**Authors:** Chin-Yuan Hsu, Yu-Pei Chan

**Affiliations:** 1 Department of Biomedical Sciences, Chang Gung University, Tao-Yuan, Taiwan; 2 Graduate Institute of Biomedical Sciences, Chang Gung University, Tao-Yuan, Taiwan; University of Crete, Greece

## Abstract

Honeybees (*Apis mellifera*) form superparamagnetic magnetite to act as a magnetoreceptor for magnetoreception. Biomineralization of superparamagnetic magnetite occurs in the iron deposition vesicles of trophocytes. Even though magnetite has been demonstrated, the mechanism of magnetite biomineralization is unknown. In this study, proteins in the iron granules and iron deposition vesicles of trophocytes were purified and identified by mass spectrometry. Antibodies against such proteins were produced. The major proteins include actin, myosin, ferritin 2, and ATP synthase. Immunolabeling and co-immunoprecipitation studies suggest that iron is stored in ferritin 2 for the purpose of forming 7.5-nm diameter iron particles and that actin-myosin-ferritin 2 may serve as a transporter system. This system, along with calcium and ATP, conveys the iron particles (ferritin) to the center of iron deposition vesicles for iron granules formation. These proteins and reactants are included in iron deposition vesicles during the formation of iron deposition vesicles from the fusion of smooth endoplasmic reticulum. A hypothetical model for magnetite biomineralization in iron deposition vesicles is proposed for honeybees.

## Introduction

Magnetite biomineralization occurs at ambient temperature, pressure, and pH in a variety of organisms, including magnetotactic bacteria [1], honeybees [2], [3], chitons [4], trouts [5], and homing pigeons [6]. One of the best understood examples of magnetite biomineralization is in magnetotactic bacteria, which carry out magnetite biomineralization in magnetosomes. Magnetotactic bacteria are a diverse group of microorganisms with the ability to use geomagnetic fields for orientation. Magnetosomes, the organelles of magnetotactic bacteria, have nanometer-sized magnetic crystals surrounded by a lipid bilayer membrane and organize into chains via a dedicated cytoskeleton within the cell [7]. Magnetosome proteins (Mms, Mps, Mam, or Mag) include approximately 30 proteins in *M. gryphiswaldense* MSR-1 and 78 proteins in *M. magneticum* AMB-1. These proteins are involved in the formation of magnetites [8], [9]. Mms 16, MpsA and Mms24 (MamA) are responsible for mediating the invagination of the cytoplasmic membrane to form magnetosomes [10]. MamJ and MamK are involved in magnetosome chain formation [7]. MagA, MamB and MamM participate in iron transport into magnetosomes. Mms6 initiates magnetite crystal formation and/or morphological regulation [11].

In honeybees, iron deposition begins on the second day after eclosion in the iron deposition vesicles (IDVs) of trophocytes. A cloudy layer just beneath the inner IDV membrane plays an important role in the formation of 7.5-nm spherical iron particles. Subsequently, iron granules (IGs) are formed by orderly aggregation of the 7.5-nm spherical iron particles in the center of the IDVs [12]. Finally, superparamagnetic magnetite (SM) is formed in the center of mature IGs [2], [3]. However, even though magnetite has been demonstrated in honeybees [2], [3], neither the proteins involved in the formation of the 7.5-nm spherical iron particles nor the proteins that convey these tiny particles to the centers of IDVs have been identified, nor has the iron deposition microenvironment of IDVs been characterized. In this study, we purified the proteins from IGs and IDVs and prepared their antibodies. We then use immunofluoresence-labeling, immunogold labeling, and co-immunoprecipitation techniques to examine the mechanism of magnetite biomineralization in the IDVs of honeybees.

## Results

### Protein purification from IGs and IDVs

**Figure 1:**
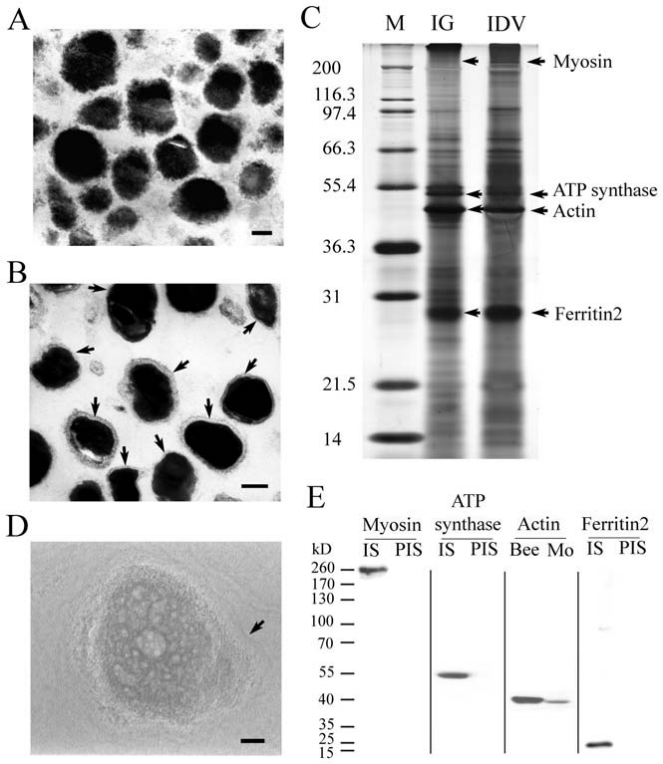
Purification and identification of proteins in IGs and IDVs and production of antibodies against these proteins. (A) TEM micrograph of purified IGs from trophocytes showing no enclosed vesicle membranes. Scale bar, 100 nm. (B) TEM micrograph of purified IDVs from trophocytes showing IGs in an enclosed vesicle membrane (arrows). Scale bar, 200 nm. (C) SDS-PAGE separation of SDS-soluble proteins from the purified IGs and IDVs. M, markers. IG, iron granule. IDV, iron deposition vesicle. Arrows denote the positions of major protein components (myosin, ATP synthase, actin and ferritin 2). (D) HRTEM micrograph of an IDV in a trophocyte of an adult worker bee. The gray gradient in the IDV indicates the existence of organic matrices. Arrow, the outer membrane of an IDV. Scale bar, 50 nm. (E) Western blot analysis to indicate the specificity of polyclonal antibodies against myosin (260 kD), ATP synthase (55 kD), actin (41 kD), and ferritin 2 (25 kD). IS, immune serum. PIS, pre-immune serum, Bee, total protein extract from worker bee trophocytes, Mo, total protein extract from mouse muscle.

To understand the mechanism of magnetite biomineralization, we purified IGs (Figure 1A) and IDVs (Figure 1B) from trophocytes according to a previously developed size-density purification procedure with only slight modifications [3] and examined them using a JEOL 2000EMII transmission electron microscope (TEM, Japan). Proteins in the IGs and IDVs were extracted by using SDS sample buffer, resolved by SDS-polyacrylamide gel electrophoresis (SDS-PAGE) (Figure 1C), and each visible protein band was excised for protein identification using MALDI-TOF mass spectrometry. From this, we identified myosin, ATP synthase, actin, and ferritin 2 as the major protein components of IGs and IDVs. These proteins in IGs and IDVs corresponded to the organic matrix that was observed in IGs and IDVs using the JEOL 4000EX high-resolution TEM (HRTEM, Japan) (Figure 1D). In addition, this result is consistent with previous study showing that ferritin is present in iron granules of honeybees [13].

### Antibodies identification and immunofluorescence assay

**Figure 2:**
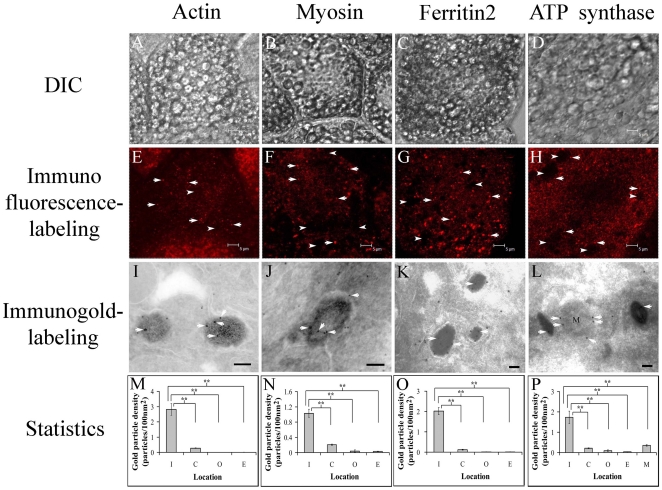
Immuno-labeling assay to detect actin, myosin, ferritin 2, and ATP synthase in IDVs. (A–D) The DIC image of trophocytes observed using CM. Scale bar, 5 µm. (E–H) Immunofluorescent detection of actin, myosin, ferritin 2, and ATP synthase in trophocytes by CM showing that all of these proteins occur in IDVs (arrows). Scale bar, 5 µm. (I–L) Immunogold-labeling detection of actin, myosin, ferritin 2, and ATP synthase in IDVs by TEM showing the presence of proteins (gold, arrows) in IDVs. Scale bar, 200 nm for actin and myosin, 100 nm for ferritin 2 and ATP synthase. (M–P) Statistical analysis of immunogold labeling assays for actin, myosin, ferritin 2, and ATP synthase in different subcellular sites. Localization of the target proteins in IDVs (I) shows a significant difference compared to the cytoplasm (C), oil vesicle (O), extracellular region (E), and mitochondria (M). Oil vesicles served as an organelle binding control. The extracellular region served as a nonspecific binding control. In all cases, the values are means ± SEM (*n* = 50), and asterisks indicate statistical significance as determined by a one-factor ANOVA (** *P*<0.01).

To examine the possible roles of these major proteins in magnetite formation, we used polyclonal antibodies against myosin, ATP synthase and ferritin 2 that were produced in-house and a commercially available polyclonal antibody against actin (BioLegend, San Diego, CA, USA; 622101) to label the locations of these proteins in IGs and IDVs. After confirming the specificity of these polyclonal antibodies to their target proteins by western blot analysis (Figure 1E), trophocytes were isolated from worker bees eight days after eclosion, fixed, and stained with these antibodies according to a standard procedure [3] for observation by confocal microscopy (CM) (Leica TCS SP2 MP). The presence of myosin, ATP synthase, actin, and ferritin 2 was verified in IDVs using an immunofluorescence assay (Figure 2A–2H). We distinguish IDVs and mitochondria under CM by comparing the images of IDVs from the stain of MTG (mitochondria dye) and the images from the stain of ferritin2 antibody (data not shown) as well as the size of IDVs (0.1–0.6 µm) [3] and mitochondria (0.5–10 µm).

### Cryosection and immunogold assay

To determine the locations of these proteins in IDVs, we stained cryosections of trophocytes with antibodies against them [14] for TEM observation. Immunogold labeling indicated that myosin, actin, and ferritin 2 were present in IGs, and ATP synthase was detected in the inner membrane of IDVs (Figure 2I–2P). The number of ATP synthase in mitochondrial was lower than that of IDVs. The reasons for this observation may be owing to: (1) only clear mitochondrial images were selected for the calculation of ATP synthase signal; (2) the images of IDVs were clearer than those of mitochondria.

### Double immunofluorescence and immunogold assay

**Figure 3:**
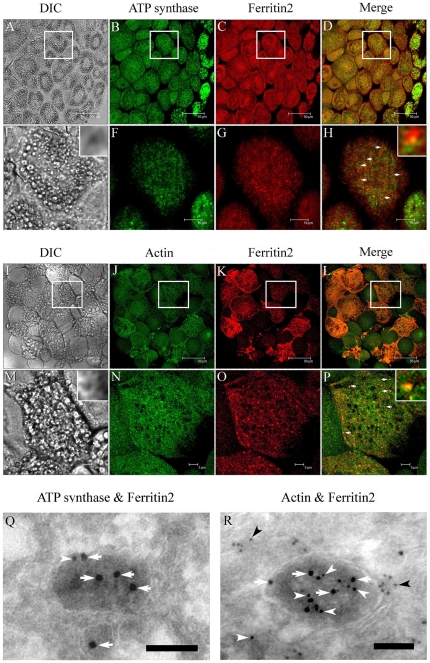
Double labeling assay of ATP synthase, ferritin 2, and actin. (A–D) ATP synthase and ferritin 2 were doubly stained and detected in IDVs (arrows) by CM. Scale bar, 50 µm. (E–H) Images enlarged from the boxed area of A–D. Scale bar, 5 µm. Insets show magnified micrographs. (I–L) Actin and ferritin 2 were doubly stained and detected in IDVs (arrows) by CM. Scale bar, 50 µm. (M–P) Images enlarged from the boxed area of I–L. Scale bar, 5 µm. Insets show magnified micrographs. (Q) Double labeling assay for detecting ATP synthase (arrowhead) and ferritin 2 (arrows) in IDVs by TEM. Scale bar, 100 nm. (R) Double labeling assay for detecting actin (arrowheads) and ferritin 2 (arrows) in IDVs by TEM. Scale bar, 100 nm.

To further confirm the co-occurrence of ATP synthase, actin, and ferritin2 in IDVs, we double stained trophocytes with antibodies against ATP synthase (MitoSciences, Eugene, Oregon, USA; ms507) and ferritin 2 for CM observation and with antibodies against actin (Chemicon, Temecula, CA, USA; mab1501) and ferritin 2 for TEM observation. The CM results showed that ATP synthase and ferritin 2 (Figure 3A–3H) and actin and ferritin 2 (Figure 3I–3P) occurred together in IDVs. The overlapping signal of actin and ferritin 2 is stronger than that of ATP synthase and ferritin 2. The TEM results also indicated the co-occurrence of ATP synthase with ferritin 2 and actin with ferritin 2 in IDVs (Figure 3Q and 3R).

### Co-immunoprecipitation assay

**Figure 4:**
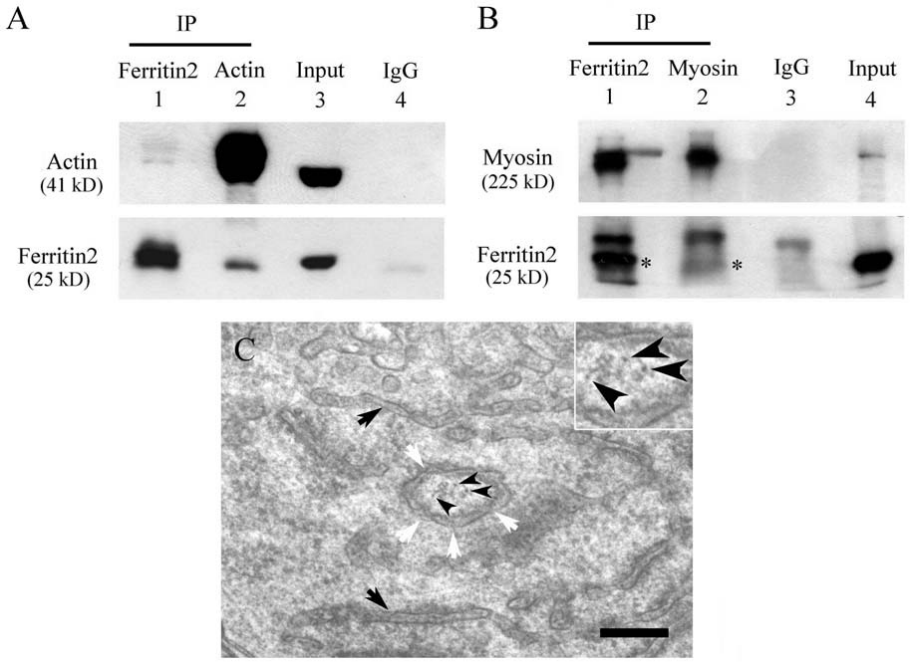
Co-immunoprecipitation assay and TEM image during the formation of an IDV. (A) Co-immunoprecipitation assay for actin and ferritin 2. Lanes 1–4: (1) IP with anti-ferritin 2 antibody; (2) IP with anti-actin antibody; (3) cell lysate input control; (4) IP with control mouse or rabbit IgG. (B) Co-immunoprecipitation assay for myosin and ferritin2. Lanes 1–4: (1) IP with anti-ferritin 2 antibody; (2) IP with anti-myosin antibody; (3) IP with control mouse or rabbit IgG; (4) cell lysate input control. * shows ferritin 2. (C) The formation of an IDV in worker bees on the second day after eclosion. The IDV derived from the fusion of fragments of SER. Inset shows magnified micrograph. Black arrow: SER. White arrow: the fusion gap of fragments of SER during IDV formation. Arrowhead: 7.5-nm diameter spherical iron particles. Scale bar, 200 nm.

Next, we investigated the interaction between actin and ferritin 2 and between myosin and ferritin 2 by co-immunoprecipitation (Co-IP) experiments. These assays indicated that actin can interact with ferritin 2 (Figure 4A) and that ferritin 2 can interact with myosin (Figure 4B), indicating that actin, myosin, and ferritin 2 can form a previously undetected complex. Therefore, it is reasonable to speculate that the actin-myosin-ferritin 2 complex may be responsible for conveying the 7.5-nm spherical iron particles to the center of IDVs in the orderly fashion that has previously been observed [12].

### IDVs derived from smooth endoplasmic reticulum (SER)

To determine the origin of IDVs, we observed the sections of trophocytes from worker bees on the second after eclosion. The result showed that IDVs were formed from the fusion of fragments of SER (Figure 4C). Primary IDVs always appear at the neighborhood of SER at the early stage of iron deposition [12]. The thickness of IDVs membrane, including an outer membrane (approx. 7.5 nm in width), a small space (approx. 10 nm in width) and an inner membrane (approx. 7.5 nm in width), is about 25 nm. This thickness of the membrane is similar to that of SER [15]. Therefore, the double membrane vesicles, IDVs, are derived from SER. Proteins and reactants for magnetite biomineralization could be included during IDVs formation.

## Discussion

### The space between the outer and inner IDV membranes is probably acidic

A previous study on magnetotactic bacteria showed that ferrous ion is present in the cytoplasm and the magnetosome in these bacteria and that this is responsible for carrying out magnetite biomineralization [10]. Additionally, magnetite was formed in an artificial vesicle containing ferrous ion when the pH was increased from 4 to 12 [16]. Therefore, we speculate that ferrous ion may also be present in the cytoplasm in honeybees for the purpose of carrying out magnetite biomineralization. Ferrous ion would then be transported into the acidic space between the outer and inner IDV membranes. This acidic space has not been determined yet. The inference of this acidic space is based on the following reasons: (1) Ferrous ion is stable in an acidic environment [16]; (2) The space accumulates a huge amount of ferrous ion but does not form iron mineral. This space is most probably acidic. At least 9×10^7^ ([(1.13×10^−13^/2.2×10^−19^)×4500]/25) molecules of ferrous ion pass through the acidic space per IDV per day before deposition in the lumen of an IDV (based on a volume of 1.13×10^−13^ ml for the lumen of an IDV with a 0.6-µm in diameter, a volume of 2.2×10^−19^ ml per 7.5-nm spherical ferritin particle, 4500 iron atoms per ferritin particle, and 25 days for iron deposition to occur) [12], [17]; (3) Iron is rapidly bound and oxidized by the ferritin particles at higher pH values, but this process is inhibited at low pH [18]. (4) Magnetite can be formed in an artificial vesicle containing ferrous ion by increasing the pH from 4 to 12 [16].

### The formation of 7.5 nm spherical iron particles in the presumed alkaline lumen of IDVs

Fe^2+^ in the acidic space is transported into the lumen of IDVs, and 2H^+^ is coordinately transported out of the lumen through a presumed H^+^/Fe^2+^ antiporter protein. Thus, the alkaline lumen of IDVs might be established and the electrolytic balance might be maintained between the lumen and the acidic space. The alkaline lumen of IDVs is speculated and has not been determined yet. In magnetotactic bacteria, a proton-driven H^+^/Fe^2+^ antiporter protein encoded by the *magA* gene has been found on the membrane of magnetosomes [19], [20]. Homologue of *magA* gene, isotig00164.Amelabd (gb|HP459622.1|), has been found in honeybees (*Apis mellifera*). The function of this H^+^/Fe^2+^ antiporter protein may be similar in magnetotactic bacteria and honeybees. Fe^2+^ enters the alkaline lumen of IDVs and is subsequently bound to ferritin, which nucleates Fe^2+^ to Fe^3+^ (Fe^2+^ gets oxidized into Fe^3+^), and these iron molecules are then hydrated into FeO (Fe^2+^), Fe_2_O_3_ (Fe^3+^), or FeOOH (Fe^3+^) [3], [18], [21]. Subsequently, ferrihydrite is reduced to form magnetite in the center of IDVs [2]. This reaction has been demonstrated in the magnetosome of magnetotactic bacteria [22], [23].

### Transporter system of magnetite biomineralization in IDVs

It has been observed that the 7.5-nm spherical iron particles (ferritin) spontaneously move to the center of IDVs in an orderly fashion [12]. This observation suggests that a regular route for ferritin transport may exist in the lumen of IDVs and a putative actin-myosin-ferritin system may play the role of the transporter in this process. An actin chain serves as the route of transport with one end of a myosin molecule being attached to the actin chain and another end to ferritin, which allows myosin to carry ferritin along the actin chain to the center of IDVs. This reaction requires Ca^2+^ and ATP, and we also identified ATP synthase in purified IGs and IDVs. This putative system could reasonably explain the function of calcium and phosphate in IGs because energy dispersive X-ray spectrum analysis showed that iron, calcium, and phosphate were present in IGs [3]. In magnetotactic bacteria, it has been demonstrated that an ATPase that is essential for iron trafficking is present in the cytoplasm [24].

### A model for magnetite biomineralization in the IDVs of honeybees

**Figure 5:**
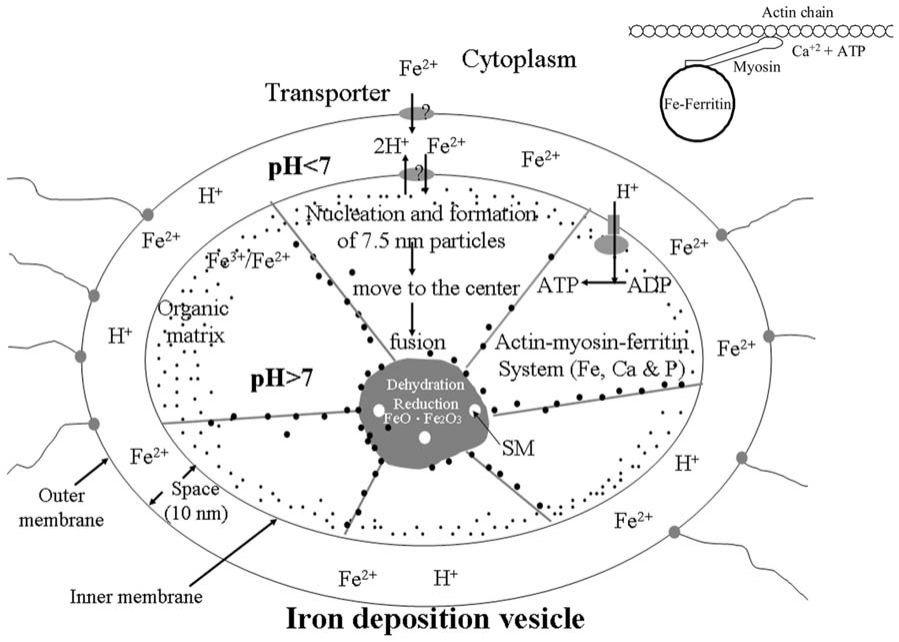
A hypothetical model of magnetite biomineralization in the IDVs of trophocytes. Inset shows the actin-myosin-ferritin transporter system. SM, superparamagnetic magnetite.

Taking all of this into consideration, we put forth the following working hypothesis for magnetite biomineralization in honeybees: Fe^2+^ from the cytoplasm is transported into the acidic space (pH<7) between the outer and inner IDV membranes via a transporter protein. An H^+^/Fe^2+^ antiporter on the inner IDV membrane then simultaneously transports one molecule of Fe^2+^ into and two molecule of H^+^ out of the acidic space to maintain a pH<7 in the acidic space and a pH>7 in the alkaline lumen of IDVs. Fe^2+^ then becomes partially oxidized to Fe^3+^, and Fe^2+^/Fe^3+^ is integrated into apoferritin in the cloudy layer of IDVs to form 7.5-nm spherical iron particles (ferritin). Ferritin attached to myosin is then transported along an actin chain to the center of IDVs in a manner that is dependent on Ca^2+^ and ATP. And finally, Fe^2+^/Fe^3+^ in the center of IDVs forms SM through oxidation-reduction and dehydration (Figure 5).

## Materials and Methods

### Honeybees

Honeybees (*Apis mellifera*) were bred in an open environment in a bee-breeding room at our institute. Sucrose and pollen grains were occasionally added to the hives as dietary supplements.

### Purification of IGs and IDVs

Purification of IGs and IDVs was carried out as previously described [3] with slight modifications. Briefly, for the purification of IGs, trophocytes from 300 adult worker bees were collected into honeybee saline containing 0.1% Triton X-100, 0.5 M sucrose, 1 M NaCl, and phenylmethylsulfonyl fluoride (PMSF)(200∶1 (v/v)) and then were homogenized with a polytron. The homogenized solution was filtered through a series of filters. The filtered solution was centrifuged against a 6.0 m solution of sucrose twice. The precipitate was collected for further analysis of IGs. For purification of IDVs, trophocytes from 300 adult worker bees were collected into honeybee saline containing 6.0 m sucrose, 1 M NaCl, and 200∶1 (v/v) PMSF and then were homogenized using a polytron. The homogenized solution was diluted with the same volume of honeybee saline and filtered through a series of filters. The filtered solution was centrifuged against a 6.0 m solution of sucrose three times. The precipitate was collected for further analysis of IDVs.

### TEM

TEM was carried out as previously described [15]. Briefly, the purified IGs, purified IDVs, or trophocytes from worker bees on the second days after eclosion were fixed in 2.5% glutaraldehyde, postfixed in 1% osmium tetroxide, dehydrated through an ethanol series, and embedded in Spurr's resin. Thin sections (60–90 nm in thickness) were cut with a diamond knife, stained with uranyl acetate and lead citrate and then examined using a JEOL JEM-2000EXII TEM (Japan) operated at an accelerated voltage of 100 kV. High-resolution TEM was performed as previously described [2] using a JEOL 4000EX HRTEM that operates at an accelerated voltage of 400 kV.

### SDS-PAGE

The purified IGs or IDVs were extracted with SDS sample buffer. After centrifugation, the extracts were resolved on an 11% SDS-gel, and the gel was stained using silver nitrate as previously described [25], [26].

### In-gel tryptic digestion and mass spectrometric analysis

In-gel tryptic digestion of silver-stained proteins and mass spectrometric analysis were carried out as previously described [25], [26]. Matrix-assisted laser desorption/ionization-time of flight (MALDI-TOF) mass spectrometric analysis was performed on an Ultraflex™ MALDI-TOF/TOF mass spectrometer (Bruker Daltonik GmbH, Bremen, Germany).

### Antibody production

Anti-myosin, anti-ferritin 2 and anti-ATP synthase antibodies were produced in rabbits using peptides corresponding to the NH_2_-terminal region of honeybee myosin (amino acids 1–17; MPKPKPQEGEDPDPTPY), the COOH-terminal region of honeybee ferritin 2 (amino acids 154–172; KIHEKANKKQDSAIAHYME), and the internal region of honeybee ATP synthase (amino acids 222–238; NQKRFNDAGEEKKKLYC). These peptides were synthesized by Kelowna International Scientific (Taipei, Taiwan). A cysteine residue was added to the NH_2_- or COOH-terminus of the peptide to facilitate coupling to BSA (as a carrier protein). The anti-peptide antibodies were affinity-purified by Sepharose 4B (GE Healthcare, Little Chalfont, Buckinghamshire, UK) coupled to the corresponding peptide. After purification, anti-peptide antibodies were concentrated and stored in 50% glycerol in PBS at −20°C. The procedures for production and affinity-purification of the anti-peptide antibodies were detailed previously [26], [27].

### Western Blotting

Workers were freshly collected from hive and dissected at 4°C. Trophocytes and fat cells from three workers were collected at 4°C and homogenized using a pestle and sonicator in 150 µl lysis buffer (C3228, Sigma, Louis, MO, USA) containing protease inhibitors (leupeptin 0.5 µg/ml, pepstatin 0.7 µg/ml, and phenylmethylsurfonyl fluoride 40 µg/ml) at 4°C. Cell extract were centrifuged at 25,000 *g* for 10 min at 4°C, and the resulting supernatant was used in Western blotting. Protein concentration was determined using protein assay reagent (#500-0006, Bio-Rad, Hercules, CA, USA). Western blotting was carried out as previously described [28]. Briefly, 30 µg of total protein was resolved on an 8–12% SDS-gel, transferred to a PVDF membrane, and then incubated with the following primary antibodies: mouse monoclonal anti-actin (1∶20,000) (Chemicon, Temecula, CA, USA; mab1501), rabbit polyclonal anti-actin (1∶5,000) (BioLegend, San Diego, CA, USA; 622101), rat monoclonal anti-myosin (1∶1,000) (Abcam, Cambridge, UK; ab51098), rabbit polyclonal anti-myosin produced in-house (1∶1,000), rabbit polyclonal anti-ferritin 2 produced in-house (1∶10,000), mouse monoclonal anti-ATP synthase (1∶15,000) (MitoSciences, Eugene, Oregon, USA; ms507), or rabbit polyclonal anti-ATP synthase produced in-house (1∶20,000). Membranes were then probed with a horseradish peroxidase-labeled secondary antibody (1∶10,000), and immunoreactive proteins were indirectly visualized by a chemiluminescence (Perkin Elmer).

### Immunofluorescence assay

An immunofluorescence assay was carried out as previously described [28]. Briefly, trophocytes and fat cells from adult worker bees were fixed with 4% paraformaldehyde in PBS for 20 min, washed with PBS three times, permeabilized with 0.2% Triton X-100 in PBS for 2 min, washed with PBS three times, and incubated with 1% bovine serum albumin (BSA) for 1 h. Cells were treated with rabbit polyclonal anti-actin (1∶50) (BioLegend, San Diego, CA, USA; 622101), rabbit polyclonal anti-myosin produced in-house (1∶300), rabbit polyclonal anti-ferritin 2 produced in-house (1∶300), or rabbit polyclonal anti-ATP synthase produced in-house (1∶250) at 4°C overnight, followed by treatment with a goat anti-rabbit IgG secondary antibody conjugated to Alexa Fluor ®594 (1∶200) (Invitrogen, Carlsbad, CA, USA; 57911A) for 1 h at room temperature. Cells were then observed at wavelengths of 590/617 nm for excitation/emission on a laser scanning CM (Leica TCS SP2 MP). A double immunofluorescence assay was performed similarly to the procedures described above. Briefly, for the ATP synthase and ferritin 2 double immunofluorescence assay, cells were treated with a mouse monoclonal anti-ATP synthase primary antibody (1∶300) (MitoSciences, Eugene, Oregon, USA; ms507), followed by treatment with a goat anti-mouse IgG secondary antibody conjugated to Alex Fluor ®488 (1∶200) (Invitrogen, Carlsbad, CA, USA; 412974). Then, cells were re-treated with the additional primary antibody rabbit polyclonal anti-ferritin 2 produced in-house (1∶300), followed by treatment with a goat anti-rabbit IgG secondary antibody conjugated to Alex Fluor ®594 (1∶200) (Invitrogen, Carlsbad, CA, USA; 57911A). Cells were then observed under wavelengths of 495/519 nm and 590/617 nm for excitation/emission on a laser scanning CM (Leica TCS SP2 MP, Germany). For the actin and ferritin 2 double immunofluorescence assay, cells were treated with mouse monoclonal anti-actin (1∶300) (Chemicon, Temecula, CA, USA; mab1501), followed by treatment with a goat anti-mouse IgG secondary antibody conjugated to Alex Fluor ®488 (1∶200) (Invitrogen, Carlsbad, CA, USA; 412974). Then, cells were re-treated with the other primary antibody, rabbit polyclonal anti-ferritin 2 produced in-house (1∶300), followed by treatment with a goat anti-rabbit IgG secondary antibody conjugated to Alex Fluor ®594 (1∶200) (Invitrogen, Carlsbad, CA, USA; 57911A). Cells were then observed under wavelengths of 495/519 nm and 590/617 nm excitation/emission on a laser scanning CM (Leica TCS SP2 MP, Germany).

### Immunogold-labeling assay

Cryo-sectioning of honeybee specimens was carried out as previously described [14]. Briefly, trophocytes and fat cells were collected from worker bees eight days after eclosion and fixed in 0.2% glutaraldehyde and 2% paraformaldehyde in phosphate buffer for 2 h. After washing out the fixation solution, cells were embedded in a 12% gelatin solution and dehydrated with l5% polyvinylpyrrolidone in 2.3 M sucrose at 4°C for 24 h. Sections 60–70 nm in thickness were cut with a diamond knife, incubated with rabbit polyclonal anti-actin (1∶10) (BioLegend, San Diego, CA, USA; 622101), rabbit polyclonal anti-myosin produced in-house (1∶10), rabbit polyclonal anti-ferritin 2 produced in-house (1∶50), or rabbit polyclonal anti-ATP synthase produced in-house (1∶50) for 30 min at room temperature. After washing several times with PBS, the sections were incubated with an anti-rabbit IgG secondary antibody conjugated to A-gold (10 nm) (1∶65), stained with uranyl acetate, and observed under TEM (JEOL 2000EMII, Japan). A double immunogold-labeling assay was performed similarly to the procedures described above. Briefly, for the ATP synthase and ferritin 2 double immunofluorescence assay, sections were treated with a mouse monoclonal anti-ATP synthase primary antibody (1∶125) (MitoSciences, Eugene, Oregon, USA; ms507), followed by treatment with a rabbit anti-mouse IgG secondary antibody (1∶500) (Jackson ImmunoResearch Laboratories, West grove, PA, USA; 77521) and a goat anti-rabbit IgG tertiary antibody conjugated to A-gold (5 nm) (1∶70). Then, sections were re-treated with the other primary antibody, rabbit polyclonal anti-ferritin 2 produced in-house (1∶125), followed by treatment with a goat anti-rabbit IgG secondary antibody conjugated to A-gold (15 nm) (1∶60). Sections were observed under TEM (JEOL 2000EMII, Japan). For the actin and ferritin 2 double immunofluorescence assay, sections were treated with a mouse monoclonal anti-actin primary antibody (1∶2,000) (Chemicon, Temecula, CA, USA; mab1501), followed by treatment with a rabbit anti-mouse IgG secondary antibody (1∶500) (Jackson ImmunoResearch Laboratories, West grove, PA, USA; 77521) and a goat anti-rabbit IgG tertiary antibody conjugated to A-gold (5 nm) (1∶70). Then, sections were retreated with the other primary antibody, rabbit polyclonal anti-ferritin 2 produced in-house (1∶300), followed by treatment with a goat anti-rabbit IgG secondary antibody conjugated to A-gold (15 nm) (1∶60). Sections were observed under TEM (JEOL 2000EMII, Japan).

### Immunoprecipitation assay

Trophocytes or fat cells were collected from three adult worker bees and lysed using 150 µl of RIPA lysis buffer (50 mM Tris-HCl, pH 7.4, 150 mM NaCl, 2.5% deoxycholic acid, 10% NP-40, 1 mM PMSF, 1 mM ethylenediaminetetraacetic acid (EDTA), 1 mM sodium orthovanadate, 1 mM sodium fluoride, 1 µg/ml aprotinin, 1 µg/ml leupeptin and 1 µg/ml pepstatin) (Upstate). 200 µl of crude cell lysates were precleared by incubating with 20 µl of protein-G agarose (Upstate) at room temperature for 1 h followed by centrifugation at 25,000 g for 10 min. The supernatants were then incubated with 1 µl of mouse monoclonal anti-actin antibody and 20 µl of protein-G agarose, 1 µl of rabbit polyclonal anti-myosin antibody and 20 µl of protein-G agarose, or 2 µl of rabbit polyclonal anti-ferritin 2 antibody and 20 µl of protein-G agarose at room temperature for 1 h, and then centrifuged at 7,000 g for 10 min. The immunoprecipitates were washed with 1 ml of RIPA buffer five times and analyzed by western blotting. 30 µg of total protein was resolved on an 8–12% SDS-gel, transferred to a PVDF membrane, and then incubated with the following primary antibodies: mouse monoclonal anti-actin (1∶20,000) (Chemicon, Temecula, CA, USA; mab1501), rabbit polyclonal anti-myosin produced in-house (1∶1,000), rabbit polyclonal anti-ferritin 2 produced in-house (1∶10,000). Membranes were then probed with a horseradish peroxidase-labeled secondary antibody (1∶10,000), and immunoreactive proteins were indirectly visualized by a chemiluminescence (Perkin Elmer).

### Statistical analysis

Differences in the mean values among the immunogold-labeling assays and western blot signals were determined by one-way ANOVA and by Tukey's HSD for pairwise comparisons. Statistical significance was set at 0.05.
